# Diversity of tick species (Acari: Ixodidae) in military training areas in Southeastern Brazil

**DOI:** 10.1590/S1984-29612022027

**Published:** 2022-05-27

**Authors:** Rubens Fabiano Soares Prado, Izabela Mesquita Araújo, Matheus Dias Cordeiro, Bruna de Azevedo Baêta, Jenevaldo Barbosa da Silva, Adivaldo Henrique da Fonseca

**Affiliations:** 1 Hospital Veterinário, Academia Militar das Agulhas Negras – AMAN, Resende, RJ, Brasil; 2 Programa de Pós-graduação em Ciências Veterinárias, Instituto de Veterinária, Universidade Federal Rural do Rio de Janeiro – UFRRJ, Seropédica, RJ Brasil; 3 Instituto de Ciências Agrárias, Universidade Federal dos Vales do Jequitinhonha e Mucuri – UFVJM, Unaí, MG, Brasil

**Keywords:** Military training, tick-borne diseases, biosecurity, zoonosis, Treinamento militar, doenças transmitidas por carrapatos, biossegurança, zoonoses

## Abstract

Tick-borne pathogens belong to one of the two main groups of occupational biohazards, and occupational exposure to such agents puts soldiers at risk of zoonotic infections, such as those caused by rickettsiae. There are few studies on acarological fauna and occupational risk in military areas in Brazil. Thus, the present study aimed to analyze the diversity of ticks present in the military training areas of municipalities in the Southeast Region of Brazil. The ticks were collected from the selected areas using the dragging and flagging techniques as well as by visual detection on the operators’ clothing, and environmental information was also recorded. A total of ten species were collected from the 66 surveyed areas, belonging to five genera and nine species: *Amblyomma sculptum*, *Amblyomma dubitatum*, *Amblyomma brasiliense*, *Amblyomma longirostre*, *Amblyomma aureolatum*, *Dermacentor nitens, Rhipicephalus* spp., *Ixodes* spp. and *Haemaphysalis* spp. The frequent presence of tick species in military training areas along with traces and sightings of wild animals, most commonly capybaras (*Hydrochoerus hydrochaeris*), in most of the studied areas, indicates high levels of exposure of the military to tick vectors of spotted fever group rickettsiae and the possible occurrence of infections among the troops.

## Resumo

As doenças transmitidas por carrapatos estão entre os dois principais grupos de riscos biológicos ocupacionais. Tal exposição ocupacional de militares os colocam sob maior risco de adquirirem doenças zoonóticas, como infecções rickettsiais, entre outras. No Brasil, há raros estudos sobre fauna acarológica e o risco ocupacional em áreas militares. Neste sentido, o presente estudo teve por objetivo analisar a diversidade de carrapatos presentes em áreas de treinamento militar de municípios da região Sudeste do Brasil. Os carrapatos foram coletados nas áreas através de arrasto de flanela, bandeiramento e/ou coleta nas vestimentas do operador, sendo registradas também informações ambientais. Das 66 áreas pesquisadas, foram coletados 9.374 carrapatos, com uma diversidade de cinco gêneros, distribuídos em dez espécies: *Amblyomma sculptum*, *Amblyomma dubitatum*, *Amblyomma brasiliense*, *Amblyomma longirostre*, *Amblyomma aureolatum*, *Amblyomma* spp., *Dermacentor nitens*, *Rhipicephalus* spp., *Ixodes* spp. e *Haemaphysalis* spp. A presença frequente de espécies de carrapatos nas áreas de instrução militar, associada aos vestígios e à presença de animais silvestres, mais comumente de capivaras na maioria das áreas estudadas, evidencia uma grande exposição dos militares a carrapatos vetores de rickettsias do Grupo da Febre Maculosa e a possível ocorrência de casos humanos nas tropas.

## Introduction

Military personnel live with the risk of physical harm or even death throughout their professional lives (Neves, 2007). Additionally, exposure to occupational hazards such as chemical, physical, and biological agents can negatively impact their health by causing illness, necessitating sick leave and reforms, and even leading to death (Silva & Santana, 2004). Outside the health arena, military personnel constitute one of the professional groups that is most frequently exposed to the greatest variety of pathogens due to the different working conditions and environments in which they conduct their training or operations (Acke et al., 2022).

Tick-borne pathogens belong to one of the two main groups of occupational biohazards (Rim & Lim, 2014). In many parts of the world, military activities result in frequent exposure to tick infestation. This occupational exposure puts military professionals at greater risk of acquiring zoonotic diseases such as rickettsial infections (Warner et al., 1996; McCall et al., 2001; Murphree et al., 2009; Premaratna et al., 2014; Weiss et al., 2019). Thus, tick-borne diseases, in addition to being a serious public health and economic problem, can be considered a matter of national security (Pages et al., 2010; Faulde et al., 2014).

The occurrence of diseases transmitted by ticks has been reported in military training environments (Sanchez et al., 1992) and military operations (Jiang et al., 2015) in the form of both isolated cases (Rooney et al., 2001; Weiss et al., 2019) and outbreaks (Petersen et al., 1989; Sanchez et al., 1992; Warner et al., 1996; Dooley & Murray, 2004; Faix et al., 2008). Therefore, the presence of ticks in environments where troops will be dispatched presents a challenge to the health-related aspects of the planning of army operations and should be considered in health surveys aimed at mitigating risks and preserving the health of the military (Kelly et al., 2002; Petersen et al., 2015).

The distribution of ticks depends on the presence of appropriate hosts as well as climatic and environmental characteristics (Szabó et al., 2013). Knowledge of the acarological fauna of a specific region can guide research on the circulation of pathogens and the occurrence of zoonoses, enabling the implementation of more effective strategies to prevent the diseases transmitted by these vectors (Petersen et al., 2015; Melo, 2018). Thus, knowledge of the species of vector ticks presents in areas where the military is trained is an important tool for risk assessment and the adoption of more effective protective measures that will mitigate the negative health impact on troops (Faulde et al., 2014; Petersen et al., 2015; Schubert & Melanson, 2020).

Little is known about the tick species present in military training areas and the occupational risk to the military of contracting diseases transmitted by ticks in Brazil. Thus, this study aimed to analyze the diversity of ticks present in areas used for military training by military organizations (MOs) in different municipalities and biomes in the Southeast Region of Brazil.

## Material and Methods

The study was conducted in 2019 between August and November, the months with the highest incidence of Brazilian spotted fever (BSF). A survey of the acarological fauna was carried out in frequently used military training areas belonging to six MOs located in the Southeast Region of Brazil.

### Inclusion criteria for tick sampling areas

For the conduction of this study, MOs of the Brazilian Army that possessed military training areas in the Southeast Region of Brazil were selected. The military units surveyed were: Três Corações, São Bento Abade, and Juiz de Fora municipalities (state of Minas Gerais); Resende, Seropédica, and Paracambi municipalities (state of Rio de Janeiro); and Campinas (state of São Paulo).

Tick collection was carried out in areas where troop movements or military training occurred. The sampling points comprised open areas in fields or forests on MO land and in a civil institutional area temporarily assigned for military training. These areas are often used to conduct training, with heavy traffic consisting of groups of military personnel during periods of use throughout the year. The number of areas sampled varied among MOs depending on the extension of each training area and the number of areas belonging to each MO.

In total, 66 areas were selected for the field collection of ticks: 14 areas in the municipalities of Três Corações and São Bento Abade, MG, belonging to the Escola de Sargento das Armas (ESA); nine areas in the municipality of Juiz de Fora, MG, belonging to the Campo de Instrução de Juiz de Fora / Centro de Educação Ambiental e Cultura (CIJF/CEAC); seven areas located in the municipalities of Seropédica and Paracambi, RJ, belonging to the Depósito Central de Munição (DCMun); 24 areas in the municipality of Resende, RJ, belonging to the Academia Militar das Agulhas Negras (AMAN); and 12 areas in the municipality of Campinas, SP, nine belonging to the 11th Brigada de Infantaria Leve (11th Bda Inf L) and three to the Escola Preparatória de Cadetes do Exército (EsPCEx), considered a single institution because they are neighboring MOs with similar characteristics (Table 1).

**Table 1:** Municipalities belonging to the Military Organizations of the Southeast Region of Brazil positive for ticks collected between August and November 2019 and their respective tick sampling points, coordinates, and elevation above sea level.

**Municipalities**	**MOs**	**Points**	**Geographic Coordinates** **(DATUM WGS84)**	**Height**
Três Corações and São Bento Abade - MG	ESA	1	21°42'26,15”S, 45°15'22,81”W	836 m
	ESA	2	21°42'21,18”S, 45°13'31,71”W	840 m
	ESA	3	21°42'19,37”S, 45°15'36,22”W	838 m
	ESA	4	21°42'07,37”S, 45°15'30,22”W	846 m
	ESA	5	21°36'11,52”S, 44°58'56,18”W	1148 m
	ESA	6	21°36'51,72”S, 44°59'49,35”W	970 m
	ESA	7	21°37'09,68”S, 44°59'34,04”W	963 m
	ESA	8	21°37'26,58”S, 44°59'09,75”W	989 m
	ESA	9	21°37'28,69”S, 44°58'45,54”W	979 m
	ESA	10	21°37'30,64”S, 44°58'13,31”W	970 m
	ESA	11	21°42'03,74”S, 45°16'43,85”W	836 m
	ESA	12	21°41'35,02”S, 45°17'08,83”W	857 m
	ESA	13	21°41'48,85”S, 45°16'38,15”W	851 m
	ESA	14	21°41'36,66”S, 45°16'42,15”W	846 m
Juiz de Fora - MG	CIJF/CEAC	1	21°42'26,32”S, 43°23'03,51”W	716 m
	CIJF/CEAC	2	21°42'10,56”S, 43°22'55,94”W	728 m
	CIJF/CEAC	3	21°42'23,73”S, 43°22'59,99”W	716 m
	CIJF/CEAC	4	21°41'35,89”S, 43°23'12,54”W	824 m
	CIJF/CEAC	5	21°41'42,92”S, 43°24'03,66”W	683 m
	CIJF/CEAC	6	21°41'42,63”S, 43°22'44,55”W	770 m
	CIJF/CEAC	7	21°42'09,18”S, 43°22'26,38”W	738 m
	CIJF/CEAC	8	21°42'27,77”S, 43°22'59,30”W	716 m
	CIJF/CEAC	9	21°42'27,89”S, 43°23'06,56”W	713 m
Paracambi and Seropédica - RJ	DCMun	1	22°39'07,12”S, 43°43'37,33”W	29 m
	DCMun	2	22°40'46,44”S, 43°44'06,63”W	44 m
	DCMun	3	22°40'50,42”S, 43°44'01,08”W	43 m
	DCMun	4	22°40'43,52”S, 43°43'49,86”W	43 m
	DCMun	5	22°39'30,88”S, 43°42'05,63”W	32 m
	DCMun	6	22°39'12,60”S, 43°42'46,89”W	41 m
	DCMun	7	22°39'07,42”S, 43°42'51,51”W	35 m
Resende - RJ	AMAN	1	22°30'36,76”S, 44°38'11,36”W	463 m
	AMAN	2	22°26'33,28”S, 44°27'12,91”W	384 m
	AMAN	3	22°26'25,71”S, 44°28'22,76”W	410 m
	AMAN	4	22°25'39,19”S, 44°28'52,53”W	433 m
	AMAN	5	22°26'08,59”S, 44°27'39,77”W	398 m
	AMAN	6	22°26'35,77”S, 44°26'41,28”W	427 m
	AMAN	7	22°24'56,20”S, 44°29'22,79”W	475 m
	AMAN	8	22°24'38,28”S, 44°29'41,55”W	494 m
	AMAN	9	22°26'23,00”S, 44°26'18,40”W	434 m
	AMAN	10	22°25'47,58”S, 44°25'25,97”W	450 m
	AMAN	11	22°26'06,82”S, 44°24'14,19”W	387 m
	AMAN	12	22°28'33,32”S, 44°25'18,51”W	382 m
	AMAN	13	22°27'00,34”S, 44°27'03,83”W	394 m
	AMAN	14	22°25'06,96”S, 44°28'09,32”W	448 m
	AMAN	15	22°23'49,47”S, 44°29'47,55”W	521 m
	AMAN	16	22°27'16,14”S, 44°28'16,75”W	437 m
	AMAN	17	22°26'44,34”S, 44°27'12,88”W	404 m
	AMAN	18	22°26'42,03”S, 44°27'23,81”W	402 m
	AMAN	19	22°26'30,52”S, 44°27'44,29”W	426 m
	AMAN	20	22°25'09,80”S, 44°28'12,40”W	406 m
	AMAN	21	22°25'03,12”S, 44°28'15,60”W	417 m
	AMAN	22	22°24'57,72”S, 44°28'08,26”W	438 m
	AMAN	23	22°25'04,08”S, 44°28'04,34”W	450 m
	AMAN	24	22°25'05,80”S, 44°28'02,40”W	437 m
Campinas - SP	EsPCEx	1	22°53'00,48”S, 47°04'41,48”W	688 m
	EsPCEx	2	22°52'56,60”S, 47°04'38,65”W	697 m
	EsPCEx	3	22°52'44,22”S, 47°04'47,06”W	666 m
	11ª Bda Inf L	4	22°52'34,94”S, 47°06'47,44”W	616 m
	11ª Bda Inf L	5	22°51'33,11”S, 47°07'04,72”W	582 m
	11ª Bda Inf L	6	22°51'17,59”S, 47°07'17,93”W	587 m
	11ª Bda Inf L	7	22°52'45,78”S, 47°05'49,73”W	627 m
	11ª Bda Inf L	8	22°52'35,13”S, 47°05'14,66”W	661 m
	11ª Bda Inf L	9	22°52'46,34”S, 47°06'52,16”W	605 m
	11ª Bda Inf L	10	22°57'51,62”S, 47°02'07,49”W	719 m
	11ª Bda Inf L	11	22°57'37,06”S, 47°02'09,62”W	712 m
	11ª Bda Inf L	12	22°57'30,23”S, 47°02'10,35”W	730 m

MOs = military organizations; ESA = Escola de Sargentos das Armas; CIJF/CEAC = Campo de Instrução de Juiz de Fora/Centro de Educação Ambiental e Cultura; DCMun = Depósito Central de Munições; AMAN = Academia Militar das Agulhas Negras; EsPCEx = Escola Preparatória de Cadetes do Exército; 11th Bda Inf L = 11th Brigada de Infantaria Leve.

All tick collection points within the sampling areas were georeferenced (Table 1), using the georeferencing application CR Campeiro 7 (C7 GPS Dados) version 3.0 for smartphones, developed by the Universidade Federal de Santa Maria, as proposed by Veiga (2016). The coordinates of the points refer to the initial regions of the sampled areas.

### Tick sampling techniques and assessment of investigated areas

Each area was sampled, for approximately 40 minutes, by the dragging method using a white flannel cloth (150 cm × 100 cm) that was passed over the undergrowth and/or the flagging technique using a piece of white cotton fabric (100 cm × 50 cm) attached to a wooden rod that was applied to shrubby or larger vegetation (Witt & Souza, 2018). Ticks found adhering to the dragging and/or flagging operators’ clothing were also collected. The operators sampled areas up to 1 m on either side of the center of the instruction areas as well as the sides of the trails and access routes leading to them. Stops were made and the flannels inspected at 10-m intervals to identify and collect any ticks adhering to the fabric.

Information about the collection points was recorded on an area information sheet containing the area number, area name, MO, coordinates, altitude, collection date, predominant vegetation type, presence of nearby watercourses, and presence (sighting) or traces (feces, tracks, and trails) of wild or domestic animals in the area.

### Taxonomic identification of ectoparasites

The ticks sighted on the clothes were collected manually or with tweezers and placed in microtubes containing isopropyl alcohol. Taxonomic identification was performed using a stereoscopic microscope (Olympus® SZX16, cellSens 1.12 program) as described by Dantas-Torres et al. (2019). Except for the larvae of *Dermacentor nitens* (as this is the only species present in Brazil belonging to genus *Dermacentor*), all other larvae were classified only at the genus level because there is no taxonomic key available for species identification in Brazil.

### Ethical and legal considerations

This study was authorized by the System of Authorization and Information on Biodiversity (SISBio; reference number 68991-1) and the Ethics Committee on the Use of Animals of the Universidade Federal Rural do Rio de Janeiro reference number 9302140819 CEUA-IV/UFRRJ).

## Results

A total of 9,374 ticks were collected from the 66 areas sampled. Their distribution is shown in Table 2. Nine tick species belonging to five genera were found, namely *Amblyomma sculptum*, *Amblyomma dubitatum*, *Amblyomma brasiliense*, *Amblyomma longirostre*, *Amblyomma aureolatum*, *Dermacentor nitens*, *Rhipicephalus* spp., *Ixodes* spp., and *Haemaphysalis* spp. The highest tick diversities were found in areas belonging to AMAN and ESA (five tick species), followed by CIJF/CEAC (four), 11th° Bda Inf L /EsPCEx (two), and DCMun (one). Of the collected ticks, 92.16% (8,639/9,374) belonged to the genus *Amblyomma*.

**Table 2:** Number of ticks collected in the Military Organization areas in the Southeast Region of Brazil between August and November 2019, by developmental stage, genus, and species.

**Genus/Stages**	**Number of ticks collected per MOs**					
	**ESA**	**CIJF**	**DCMun**	**AMAN**	**11th BdaInfL/EsPCEx**	**Total**
*Amblyomma* spp. larvae	116	213	204	1626	2083	4242
*Amblyomma sculptum* – nymph	788	256	292	437	2407	4180
*Amblyomma sculptum* – adult	8	0	2	8	104	122
*Amblyomma dubitatum* – nymph	3	28	0	3	1	35
*Amblyomma dubitatum* – adult	0	1	0	3	9	13
*Amblyomma brasiliense* – nymph	0	0	0	42	0	42
*Amblyomma brasiliense* – adult	0	0	0	1	0	1
*Amblyomma longirostre* – nymph	0	0	0	2	0	2
*Amblyomma aureolatum –* nymph	2	0	0	0	0	2
*Dermacentor nitens* – larvae	43	0	0	554	0	597
*Rhipicephalus* spp. *–* larvae	120	0	0	0	0	120
*Ixodes* spp. – larvae	0	5	0	0	0	5
*Haemaphysalis* spp. – larvae	0	13	0	0	0	13
**Total**	1080	516	498	2679	4604	9374

MOs = military organizations; ESA = Escola de Sargentos das Armas; CIJF = Campo de Instrução de Juiz de Fora/Centro de Educação Ambiental e Cultura; DCMun = Depósito Central de Munições; AMAN = Academia Militar das Agulhas Negras; EsPCEx = Escola Preparatória de Cadetes do Exército; 11th Bda Inf L = 11th Brigada de Infantaria Leve.

### Percentage of tick-positive areas

Ticks were found in the training grounds of almost all MOs. Of the 66 areas sampled, 89.39% (59/66) tested positive for the presence of ticks in the environment, while the presence of ticks was not detected in 10.61% (7/66) with the sampling techniques used in the study. The percentage of tick-positive MOs areas, distributed by the developmental stage, genus, and species of the collected ticks, is listed in Table 3.

**Table 3:** Percentage of areas belonging to the Military Organizations of the Southeast Region of Brazil positive for ticks collected between August and November 2019, by developmental stage, genus, and species.

**Genus/Stages**	**Percentage of positive areas for ticks in MOs** **(Nr positive areas/Nr total areas)**					**Total %**
	**ESA**	**CIJF**	**DCMun**	**AMAN**	**11th BdaInfL** **/EsPCEx**	
Family Ixodidae	100% (14/14)	77.78% (7/9)	85.71% (6/7)	83.33% (20/24)	100% (12/12)	89.39% (59/66)
Genus *Amblyomma*	100% (14/14)	77.78% (7/9)	85.71% (6/7)	75% (18/24)	100% (12/12)	86.36% (57/66)
*Amblyomma* spp. larvae	42.86% (6/14)	66.67% (6/9)	28.57% (2/7)	16.67% (4/24)	83.33% (10/12)	42.42% (28/66)
*A. sculptum* - nymph	100% (14/14)	77.78% (7/9)	85.71% (6/7)	45.83 (11/24)	100% (12/12)	75.76% (50/66)
*A. sculptum* - adult	35.71% (5/14)	0% (0/9)	28.57% (2/7)	16.67% (4/24)	66.67% (8/12)	28.79% (19/66)
*A. dubitatum* - nymph	14.26% (2/14)	33.33% (3/9)	0%	8.33% (2/24)	8.33% (1/12)	12.2% (8/66)
*A. dubitatum* - adult	0%	11.11% (1/9)	0%	12.50% (3/24)	25% (3/12)	10.61% (7/66)
*A. brasiliense* - nymph	0%	0%	0%	37.50% (9/24)	0%	13.64% (9/66)
*A. brasiliense* - adult	0%	0%	0%	4.17% (1/24)	0%	1.52% (1/66)
*A. longirostre* - nymph	0%	0%	0%	8.33% (2/24)	0%	3.03% (2/66)
*A. aureolatum* - nymph	14.29% (2/14)	0%	0%	0%	0%	3.03% (2/66)
*Dermacentor nitens* - larvae	7.14% (1/14)	0%	0%	20.83% (5/24)	0%	9.09% (6/66)
*Rhipicephalus* spp. *-* larvae	14.29% (2/14)	0%	0%	0%	0%	3.03% (2/66)
*Ixodes* spp. - larvae	0%	33.33% (3/9)	0%	0%	0%	4.55% (3/66)
*Haemaphysalis* spp. – larvae	0%	11.11% (1/9)	0%	0%	0%	1.52% (1/66)

Nr = number; MOs = military organizations; ESA = Escola de Sargentos das Armas; CIJF = Campo de Instrução de Juiz de Fora e Centro de Educação Ambiental e Cultura; DCMun = Depósito Central de Munições; AMAN = Academia Militar das Agulhas Negras; EsPCEx = Escola Preparatória de Cadetes do Exército; 11th Bda Inf L = 11th Brigada de Infantaria Leve.

### Evaluation of the investigated areas

The presence of ticks (that can occasionally be infected with agents pathogenic for humans), environmental anthropization (as indicated by the presence of pastures and forest fragmentation), the transit of military personnel in training areas, and the presence of host animals that act as reservoirs for pathogenic agents (mainly capybaras [*Hydrochoerus hydrochaeris*]) and vector carriers (horses and canines for military use) in the MO areas were recorded.

Traces of capybaras were found in 53% (35/66) of the surveyed areas, all of which were close to watercourses (rivers, streams, or lakes), with vegetation characterized by lawns, dirty fields, forest fragments, or ciliary forests.

Relating to the presence of *A. sculptum*, of the 51 areas where this tick species was detected, in 66.67% (34/51) of them, the presence of capybaras (footprints, feces or sightings of these animals) was also observed at the time of sampling. In DCMun and 11th Bda Inf L/EsPCEx, the presence of capybaras was observed in 100% of the sampling areas where *A. sculptum* was detected (6/6 and 12/12 areas, respectively).

## Discussion

The present study was the first major acarological survey carried out in different MO areas in Brazil. Most of the ticks collected from the 66 sampled areas belonged to the genus *Amblyomma*, the main vector of zoonotic diseases in Brazil (Szabó et al., 2013). Of the nine species identified in the MO areas, four are commonly observed to affect humans who enter infested environments: *A. sculptum*, *A. aureolatum*, *A. brasiliense*, and *A. dubitatum* (Guglielmone et al., 2006). *Amblyomma sculptum* and *A. aureolatum* are the main vectors of one of the most lethal rickettsiosis in the world, i.e., Brazilian spotted fever (BSF) caused by *Rickettsia rickettsii* (Labruna et al., 2011a). Furthermore, although *A. longirostre*, *Dermacentor* spp., *Haemaphysalis* spp., *Ixodes* spp., and *Rhipicephalu*s spp. are considered non-anthropophilic, they can bite humans and are potential vectors of zoonoses, requiring further research (Labruna et al., 2005; Guglielmone et al., 2006; Labruna et al., 2007; Hun et al., 2008; Serra-Freire et al., 2011; Reck et al., 2018; Carvalho et al., 2020; Flores et al., 2020; Muñoz-Leal et al., 2020; Félix et al., 2021).

The most frequent tick species in this study was *A. sculptum*, representing 45.89% (4,302/9,374) of all ticks sampled. This species has a wide distribution from the south to the north of Brazil except for the Amazon Forest and Rio Grande do Sul. The distribution of *A. sculptum* seems to be linked to the tropical climate as it is present mainly in the Cerrado, Pantanal, and degraded areas of the Atlantic Forest, whereas it has not been detected in well-preserved and natural remnants of this biome (Szabó et al., 2006; Szabó et al., 2009; Estrada-Peña et al., 2014; Krawczak, 2016; Martins et al., 2016). The biomes researched in this study comprised areas of the Cerrado or Atlantic Forest (already degraded or fragmented), fields, riparian forests, and anthropized rural and peri-urban areas, environments where this species was also found to predominate in other studies (Souza et al., 2006; Szabó et al., 2007, 2013, 2018; Silveira & Fonseca, 2013; Brites-Neto et al., 2015; Barbieri, 2016; Melo, 2018; Pajuaba et al., 2018).

*Amblyomma sculptum* is the tick that most frequently parasitizes humans, being the predominant species found in studies in Southeastern Brazil (Lemos et al., 1997; Labruna, 2009; Szabó et al., 2013, 2018; Franco, 2018). It is the main vector of BSF, a disease whose incidence, mortality rate, and case severity have increased, particularly in the Southeast Region of Brazil (Oliveira et al., 2016). The predominance of the vector in the surveyed military areas of Southeastern Brazil corroborates the abovementioned findings. Especially in the municipality of Campinas, SP, the high number of *A. sculptum* specimens collected in the areas belonging to the 11th Bda Inf L /EsPCEx confirms previous studies indicating severe infestations of *A. sculptum* in this municipality (Franco, 2018) and highlights the high risk of BSF transmission in this locality. A predominance of *A. sculptum* nymphs was found in all MO areas surveyed except for AMAN. Several studies point to seasonality in the developmental stages of *A. sculptum*, with the larval and nymphal stages occurring at the time of year when the samples were collected, i.e., August to November (Lemos et al., 1997; Oliveira et al., 2000; Labruna et al., 2002; Souza et al., 2006; Silveira & Fonseca, 2013; Brites-Neto et al., 2015; Barbieri, 2016). Although all the MO areas studied are in the Southeast Region, differences in environmental conditions such as microclimate, vegetation cover, and anthropic activities, in addition to the diversity and activity of tick hosts, may contribute to seasonal variations in the stages of *Amblyomma* spp. present in each area (Silveira & Fonseca, 2013).

These findings also highlight the increased risk of disease transmission faced by the military at specific times of the year when using the MO areas. The highest numbers of BSF cases and deaths in the Southeast Region occur mainly between September and November, a fact that is associated with population peaks of *A. sculptum* nymphs in the environment, the developmental stage that most commonly transmits *R. rickettsii* to humans in the region (Lemos et al., 1997; Szabó et al., 2013; Souza et al., 2015; Araújo et al., 2016; Oliveira et al., 2016).

*Amblyomma dubitatum* can infest humans, but its role as a vector of BSF is still unclear (Labruna, 2009). Although *A. dubitatum* can transmit *R. rickettsii* under laboratory conditions (Sakai et al., 2014), it is not a frequent vector in regions where the disease is endemic because it is less aggressive than other species (Pajuaba et al., 2018), with the infestation of humans by this species being an unusual occurrence (Labruna et al., 2007; Sakai et al., 2014).

In addition, the spatial distribution of *A. dubitatum* in the environment is restricted due to factors related to its ecology and behavior, such as its preference for wetter microhabitats and its limited hunting and ambush behavior (Szabó et al., 2013; Barbieri, 2016; Pajuaba et al., 2018).

*Amblyomma aureolatum* has been found in higher-altitude areas of the Atlantic Forest, being more frequent in localities over 700 m above sea level (Sabatini et al., 2010). Corroborating these observations, *A. aureolatum* was found in the present study in Três Corações and São Bento Abade, MG, which are transition regions between the Atlantic Forest and Cerrado, in georeferenced sampling areas at 836 m and 963 m above sea level.

Carnivores are the primary hosts of *A. aureolatum* in its adult stage, whereas the larval and nymphal stages occur in passerine birds and small rodents (Pinter et al., 2004; Guglielmone et al., 2006; Szabó et al., 2009). The adult forms of these ticks have been found in domestic dogs from rural areas and those that frequent rural or peri-urban forest areas, with increasing reports of infestations in dogs in Brazil. This tick is a competent vector of *R. rickettsii* (Labruna et al., 2011b) and is the main vector for BSF in the metropolitan region of São Paulo, where dogs are responsible for bringing ticks into contact with humans, thereby contributing to the ecoepidemiology of the disease (Szabó et al., 2013).

According to Saraiva et al. (2014), unfed nymphs and unfed adult male ticks of the species *A. aureolatum* needed to attach to the host for 10 hours to successfully transmit a virulent strain of *R. rickettsii*. In contrast, partially fed adults needed only up to 10 minutes of attachment for transmission of *R. rickettsii* to the host. A review on Rocky Mountain spotted fever reported that an infected tick required a minimum feeding period of 2 to 10 hours to transmit *R. rickettsii* to humans (Biggs et al., 2016). In Brazil, studies found that *A. sculptum* (cited as *A. cajennense*) adult ticks infected with *R. rickettsii* required 36 hours of feeding to transmit the agent to guinea pigs (Magalhães, 1957; Saraiva et al., 2014). However, to date, there are no publications that describe similar experiments analyzing the time required for the transmission of *R. rickettsii* by nymphs and adults, both fed and unfed, of other ticks of the genus *Amblyomma*, such as *A. sculptum*. Nevertheless, there are indications that different genera and species of ticks require different feeding times for the transmission of the bacteria depending on transmission potentials and physiological variations, such as the production and composition of saliva, specific to each species (Moore, 1911; Ricketts, 1991; Spencer & Parker, 1923; Saraiva et al., 2014; Esteves et al., 2017). It should be noted that *R. rickettsii* is transmitted differently by *A. aureolatum* and *A. sculptum*, with two epidemiological scenarios for the resulting disease in the southeast of Brazil (Angerami et al, 2012; Szabó et al., 2013; Saraiva et al., 2014). The reason for this difference is that *A. aureolatum* ticks, which are generally carried by dogs to the humans they infest, have already ingested a blood meal while on the animals and activated the rickettsiae for infection. Thus, the activated rickettsiae can be transmitted rapidly to the human host once the ticks are attached (Saraiva et al., 2014). Additionally, the strain of *R. rickettsii* transmitted by *A. aureolatum* appears to be more virulent, with a faster and more severe clinical course and higher mortality rates (Angerami et al., 2012).

Four of the MOs involved in the study (ESA, AMAN, 11th Bda Inf L, and DCMun) had military working dogs, with War Dogs Sections within the military areas. Cunha et al. (2014) studied the epidemiology of BSF in the municipality of Resende, RJ, and observed that dogs that frequented pastures and forests were almost three times more likely to be seropositive for rickettsiae of the spotted fever group (SFG), thereby functioning as important sentinels of the disease.

In this regard, the detection of *A. sculptum* and *A. aureolatum* in military instruction areas indicates that military working dogs, when used in training or operations, may become infested with ticks and act as carriers, bringing them into close contact with military personnel and increasing the risk of illness. Furthermore, dogs may also act as rickettsiae amplifiers as they commonly harbor another tick species, *Rhipicephalus sanguineus* (Piranda et al., 2011).

*Amblyomma brasiliense* was found in the Atlantic Forest areas studied. The main hosts of this tick species are collared peccaries (*Dicotyles tajacu*) and peccaries (*Tayassu pecari*), although it is found on other hosts (Szabó et al., 2009). This species also infests humans, and the larvae and nymphs are considered one of the most aggressive to humans in Brazil (Guglielmone et al., 2006; Szabó et al., 2006; Valente et al., 2020).

Although the role of *A. brasiliense* in the transmission of zoonoses to humans is not well understood, a new trypanosomatid pathogen named *Trypanosoma amblyommi* was identified and isolated from *A. brasiliense* ticks in Itatiaia National Park (Marotta et al., 2018). The discovery of this pathogen of still unknown zoonotic potential calls attention to the possible emergence of diseases transmitted by this tick species. In the present study, *A. brasiliense* was collected only in the municipality of Resende, RJ, in Atlantic Forest fragments of the AMAN instruction field in a buffer area adjacent to the INP where military instruction is conducted.

*Amblyomma longirostre* is a tick that mainly parasitizes birds in its larval and nymphal stages and is commonly found in hedgehogs (*Sphiggurus spinosus* and *Sphiggurus villosus*) in its adult form (Guglielmone et al., 2006). Human parasitism by this tick, although rare, has been reported in Brazil (Reck et al., 2018; Valente et al., 2020). This tick is commonly found to be infected by *Rickettsia amblyommatis* (Abreu et al., 2019), but the pathogenicity of this agent and the role of this tick as a vector of diseases in humans remain unknown, requiring further studies.

In Asia, Europe, and North America, ticks of the genus *Haemaphysalis* have had a serious impact on human and animal health (Pritt, 2020; Dwużnik-Szarek et al., 2021). In Central America, *R. rickettsii* has been detected in human patients with spotted fever and *Haemaphysalis leporispalustris* ticks collected from wild hares (*Sylvilagus brasiliensis*), suggesting the possible involvement of this genus of ticks in the ecoepidemiology of the disease (Hun et al., 2008). In South America, GFM rickettsiae have also been detected in this tick genus (Labruna et al., 2005; Labruna et al., 2007). *Sylvilagus brasiliensis* is an abundant animal in regions where BSF is endemic in Southeastern Brazil and is cited as a potential amplifying host of *R. rickettsii* with possible involvement in the epidemiology of the disease (Labruna, 2013).

The genus *Ixodes* is also an important vector of zoonotic diseases, mainly borreliosis, in North America and Europe (Marques et al., 2021). However, in South America, it is reportedly rare to record this genus parasitizing humans (Guglielmone et al., 2006). In Brazil, the specie *Ixodes loricatus* is commonly found parasitizing didelphids, with only one report of human bited by this tick specie (Serra-Freire et al., 2011).

Nevertheless, recent studies have identified potential zoonotic agents in *Ixodes* ticks in Brazil and neighboring countries (Carvalho et al., 2020; Flores et al., 2020; Muñoz-Leal et al., 2020; Félix et al., 2021). A spirochete, *Borrelia burgdorferi* sensu lato (Bbsl), was recently detected in *Ixodes paranaensis* ticks collected in Ibitipoca State Park, MG. The spirochete *“Candidatus* Borrelia ibitipoquensis*”* is phylogenetically very close to *Borrelia valaisiana*, recognized as a zoonosis transmitted by *Ixodes* ticks in Eurasia (Muñoz-Leal et al., 2020).

Although *Dermacentor nitens* and *Rhipicephalus microplus* are monoxenous ticks considered to be highly specific to their hosts, they have been sporadically reported to infest humans in South America, including Brazil (Guglielmone et al., 2006; Reck et al., 2018; Szabó et al., 2020). Seroepidemiological evidence indicates that occupational exposure to such ticks may favor the occurrence of diseases in humans (Eraso-Cadena et al., 2018). Bioagents with zoonotic potential such as *Borrelia*, *Rickettsia*, and viruses have been detected in such ticks, indicating the possibility of their acting as vectors of diseases in humans (Gonçalves et al., 2014; Cordeiro et al., 2018; Oliveira Pascoal et al., 2019; Oliveira et al., 2020).

Regarding the environmental assessment of all the MOs areas studied, it is worth mentioning that in the Southeast Region of Brazil, in areas where BSF is endemic, the capybara is the main host responsible for maintaining the populations and high environmental densities of *A. sculptum* as well as the amplification of rickettsial infections in tick populations (Labruna, 2009; Souza et al., 2009; Queirogas et al., 2012; Szabó et al., 2013; Labruna, 2013). As it is a reservoir of rickettsiae (Souza et al., 2009; Labruna, 2013), its presence in areas where the vector is also found has been associated with a greater risk of pathogen circulation and disease occurrence in humans (Labruna, 2009; Szabó et al., 2013; Souza et al., 2015). Thus, the high levels of tick infestation and the presence and/or traces of capybaras in the MOs studied indicate a potential risk of military personnel being exposed to tick vectors of SFG rickettsiae and the possible occurrence of disease in humans in these military areas.

Labruna (2013) stated that the occurrence of BSF cases, like that of other vector-borne diseases, is directly related to the size of the tick population. Human infestation by *Amblyomma* spp. is an accidental event, resulting from the large number of free-living ticks in the environment.

The findings of the present study reveal epidemiological conditions that may favor the occurrence of diseases transmitted by ticks, such as BSF, in military areas, as already warned of in other studies (Silveira, 2010; Dantas-Torres et al., 2012; Melo, 2018). The presence of vector ticks, amplifying hosts, and favorable habitats in military areas may contribute to an increased risk of exposure to highly lethal zoonoses (Labruna, 2009; Szabó et al., 2013; Souza et al., 2015). Thus, this study demonstrates the existence of occupational risk of exposure to diseases transmitted by ticks among military personnel working in the studied areas, with a potential impact on their health.

The fact that the researched areas are within regions where BSF is endemic or cases have been confirmed (Brasil, 2007), along with recent research suggesting the possibility of the emergence of new pathogens and vectors in Brazil and worldwide, makes evident the need for biosecurity measures to protect the health of the military as well as greater awareness of the subject among health professionals and managers of the Armed Forces at all levels. As part of the collective protective measures, it is imperative to establish an acarological and epidemiological surveillance program for BSF in the institutional areas studied, as proposed by the Joint Resolution SMA/SES Nº 01 of July 1, 2016 (São Paulo, 2016). Taking this resolution as an example, the data from the present study classify such areas as “predisposed areas” to the occurrence of infection cases in humans. Thus, there is a need for further serological research on sentinel animals and constant acarological monitoring to better define the risk and appropriately classify areas, which would guide the management of the existing health risk in each of the MO areas more effectively.

The Ministry of Defense has shown concern about the human dimension and health in the Brazilian Armed Forces (Brasil, 2020a, 2020b), with the prevention of occupational biohazards, such as the risk of diseases transmitted by ticks, being a strategic factor for the protection of the health of the Forces that should be carefully considered (Faulde et al., 2014; Petersen et al., 2015). Research, health education, and the consistent use of collective and individual protection measures by the military can contribute to preventing and mitigating such occupational risks (Schubert & Melanson, 2020).
